# Association between screen time and self-reported balance disorders in middle-aged and older adults: national health and nutrition examination survey

**DOI:** 10.1007/s40520-024-02778-8

**Published:** 2024-06-10

**Authors:** Minjun Fu, Lingju Zhang, Xiaoyu Zhao, Zhijun Lv, Pei Tang

**Affiliations:** Center for Rehabilitation Medicine, Department of Neuro-electrophysiology, Zhejiang Provincial People’s Hospital (Affiliated People’s Hospital), Hangzhou Medical College, Hangzhou, Zhejiang 310014 China

**Keywords:** Screen time, Balance disorders, Adults, Television, Video games

## Abstract

**Background:**

Balance disorders can give rise to sensations of instability, lightheadedness, vertigo, disequilibrium, or syncope, ultimately leading to grave medical, physical, emotional, and societal ramifications. These conditions are highly prevalent among individuals aged 40 and above. Screen time encompasses activities associated with television viewing, video game playing, and non-work-related computer usage. Prolonged screen exposure may engender a spectrum of health issues and even elevate overall mortality rates. However, the available evidence on the potential link between excessive screen time and balance dysfunction remains limited.

**Aims:**

The primary aim of this study was to explore the possible association between prolonged screen exposure and impaired balance function.

**Methods:**

This cross-sectional study utilized data from participants who completed a comprehensive questionnaire in the NHANES database between 1999 and 2002, all of whom were aged over 40 and under 85 years. Participants’ screen time was categorized into two groups (< 4 h/d and  ≥4 h/d) for subsequent data analysis. Logistic regression, combined with propensity score matching (PSM), was employed to investigate the correlation between screen time and balance disorders.

**Results:**

A total of 5176 participants were enrolled in this study, comprising 2,586 men and 2,590 women, with a prevalence rate of balance disorders at 25.7% (1331/5176). The incidence of balance disorders was found to be significantly higher among individuals who spent 4 hours or more per day on screen time compared to those with less screen time (*P*<0.001). Multivariate logistic analysis conducted on the unmatched cohort revealed a significant association between screen time and balance disorders, with an odds ratio (OR) 1.8 (95%CI 1.57 ∼ 2.05). These findings remained consistent even after adjusting for confounding factors, yielding an OR 1.43 (95%CI 1.24 ∼ 1.66). Moreover, the association persisted when employing various multivariate analyses such as propensity score matching adjusted model, standardized mortality ratio weighting model and pairwise algorithmic model; all resulting in ORs ranging from 1.38 to 1.43 and p-values < 0.001.

**Conclusions:**

After controlling for all covariates, screen time (watching TV, playing video games, and using computers outside of work) was associated with balance dysfunction among middle-aged and older adults. This finding may offer a possible idea for the prevention of dizziness and balance disorders. Nevertheless, additional research is imperative to further validate these results.

**Supplementary Information:**

The online version contains supplementary material available at 10.1007/s40520-024-02778-8.

## Introduction

Balance disorders can arise from aberrations in one or multiple physiological systems, encompassing the visual, proprioceptive, and vestibular domains. These abnormalities may give rise to sensations of instability, dizziness, vertigo, postural imbalance, or syncope [1]. It has been reported that the annual prevalence of balance impairments among middle-aged and elderly individuals ranges from 20 to 50%, with a significant number of cases being chronic in nature [2–4]. These conditions are often accompanied by symptoms such as nausea, instability, sleep disturbances, disruption of daily activities, and emotional distress, significantly impacting the well-being of patients [5, 6]. Studies have demonstrated that balance disorders are associated with an increased risk of cardiovascular disease, cancer, and all-cause mortality [7]. Given the substantial prevalence and detrimental impact of this issue on individuals’ health, it is imperative to allocate attention towards addressing it in a more specialized and academic manner.

The term “screen time” encompasses activities associated with television viewing, video game playing, and computer usage [8]. Given the advancements in technology, screen time has become an integral aspect of daily routines. It is widely acknowledged that excessive exposure to screens can result in ocular strain, visual impairment, dryness of the eyes, headaches, and personal discomfort, consequently leading to sleep disturbances and mood disorders. Numerous studies have consistently established a strong association between screen time and an elevated susceptibility to sleep disturbances, mood disorders, and various other health complications [9–12]. However, no research has yet explored the association between screen time and balance disorders. Therefore, this study postulated a hypothesis and subsequently substantiated the correlation between these two variables.

## Methods

The data for this cross-sectional study were obtained from NHANES (1999 to 2000,2001 to 2002 cycles), a survey by the Centers for Disease Control and Prevention. The objective of the NHANES project was to assess the health and nutritional status of non-institutionalized Americans using a stratified multistage probability survey. By conducting home visits, screening, and laboratory tests through a mobile examination center (MEC), NHANES collected detailed demographic and health information. The NHANES received approval from the Ethics Review Committee of the National Center for Health Statistics (NCHS), and all participants provided written informed consent forms before participation. This is a secondary analysis, no additional approval from the Institutional Review Board was required. The NHANES data can be accessed through the official NHANES website (http://www.cdc.gov/nchs/nhanes.htm).

We determined whether a participant experienced balance disorders based on their replies to the question in the balance section questionnaire: “During the past 12 months, have you had dizziness, difficulty with balance or difficulty with falling?” Screen time behavior was measured using the question “Over the past 30 days, on a typical day how much time altogether did you spend on a typical day sitting and watching TV or videos or using a computer outside of work?” According to the obtained data, we conducted a preliminary regression analysis on an hourly basis to determine the inflection point, in addition to referring to some literature. Finally, screen time was categorized in categories (< 4 h/day and ≥4 h/day) for data analysis [13, 14].

Race was categorized as non-Hispanic white, non-Hispanic black, Mexican American, or other races. According to the educational level data provided by the NHANES database, the educational level was categorized as less than 9 years, 9 to 12 years, and more than 12 years, which respectively represent less than high school, high school or equivalent, and college or above education. According to a US government report, marital status was classified as married, living with a partner, or living alone. Family income was categorized into three groups based on the poverty income ratio (PIR): low (PIR *≤* 1.3), medium (PIR > 1.3 to 3.5), and high (PIR > 3.5) [7]. Calorie intake obtained from participants’ 24-h nutritional information. Total cholesterol levels were measured enzymatically. The determination of previous diseases (hypertension, diabetes, stroke, and coronary heart disease) were based on the inquiry in the questionnaire of whether the doctor had been informed of the condition in the past. According to preceding literature definitions, smoking status was categorized as never smokers (smoked less than 100 cigarettes), current smokers, and former smokers (quit smoking after smoking more than 100 cigarettes). Alcohol drinking status was determined if participants had consumed at least 12 drinks of any type of alcoholic beverage in any one year. Physical activity was classified as light (Low-intensity activities are good for health, but do not result in sweating or faster breathing), moderate (at least 10 min of activity, resulting in only light sweating or a mild to moderate increase in breathing or heart rate), and vigorous (at least 10 min of activity, resulting in profuse sweating or an increase in breathing or heart rate).

This is a secondary examination of publicly accessible datasets. Categorical variables were represented by proportions (%) while continuous variables were described by the mean (standard deviation, SD) or median (interquartile range, IQR), as appropriate. Normally distributed continuous variables were compared by using two-sided Student t tests. Continuous variables, which were not normally distributed, were compared by using the Wilcoxon rank test. Two-sided likelihood ratio chi-square tests or Fisher exact test were used to compare categorical variables. Logistic regression models were used to determine the odds ratios (ORs) and 95% confidence intervals (95% CIs) for the relationship between screen time and balance disorders. The models were enhanced by incorporating variables with a univariate analysis P value less than 0.1, variables exhibiting a change in effect value greater than 10%, and covariates of clinical significance reported in previous studies to ensure the stability and robustness of the results. In the concrete analysis, Model1 was adjusted for sociodemographic characteristics, including age, sex, race, marital status, education level, and family income. Model2 was adjusted for sociodemographic characteristics and previous diseases (hypertension, diabetes, stroke, and coronary heart disease). Model3 was adjusted for sociodemographic characteristics, previous diseases as well as smoking status and alcohol drinking. Model4 was a fully adjusted model that includes all covariates from the above models along with previously reported clinically significant covariates (calorie intake, total cholesterol levels, and physical activity).

Propensity score matching (PSM) was used to adjust for confounding factors and improve comparability between groups in this study. A 1:1 nearest neighbor matching algorithm was applied and a caliper width was 0.2. Age, sex, race, education level, marital status, family income, calorie intake, total cholesterol, hypertension, diabetes, stroke, and coronary heart disease, smoking statue, alcohol drinking and physical activity were adjusted by the logistic regression model to calculate the propensity score. The degree of PSM was estimated using standardized mean differences (SMDs). Optimal balance on a parameter is generally achieved when the SMD is equal to or below 0.1. The standardized mortality ratio weighting (SMRW) [15], and Pairwise algorithmic (PA) [16] model, that unified the distribution of the risk factors for both groups was used to confirm the robustness of the results.

Interaction and stratified analyses were conducted according to age, sex, family income, education level, physical activity, smoking status and alcohol drinking.

All data were processed using the Free Statistics software version 1.9.

## Results

**Fig. 1:**
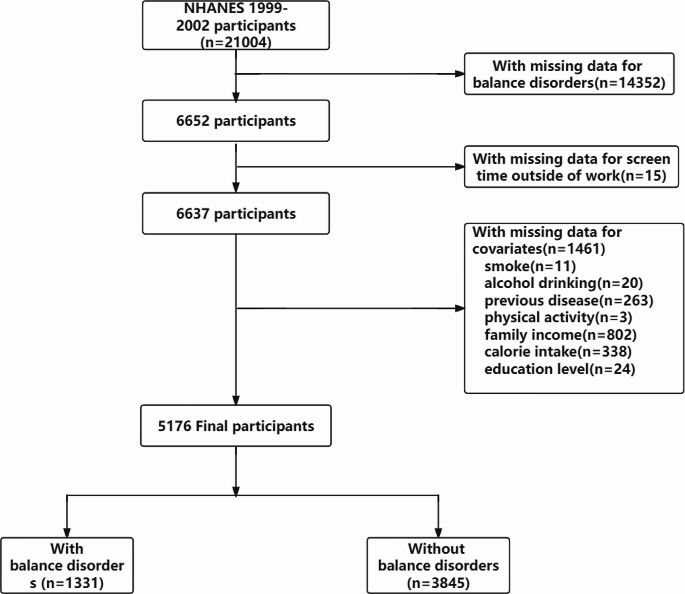
Flowchart of participants selection

Ultimately, this cross-sectional study included 5176 participants from the NHANES between 1999 and 2002 in the analysis, 1,331 (25.7%) had a balance disorder. The detailed inclusion and exclusion process is shown in Fig. 1.

The characteristics of all participants are listed in Table 1. The age of all participants was 60.6 ± 13.4 years, and the age range is 40–85 years. The proportion of men to women was roughly equal. Most patients were Non-Hispanic white. Participants with increased screen time exhibited a higher likelihood of comorbidities such as hypertension, diabetes, coronary heart disease, and stroke; reported lower levels of physical activity; had a decreased household income; and demonstrated a propensity for smoking and living alone.

**Table 1:** >Characteristics of participants

Variables	Total	screen time < 4 h	screen time ≥4 h		*p* value
	(*n* = 5176)	(*n* = 3754)		(*n* = 1422)	
**Sex**, n (%)					0.643
Male	2586 (50.0)	1883 (50.2)		703 (49.4)	
Female	2590 (50.0)	1871 (49.8)		719 (50.6)	
**Age**(year), Mean (SD)	60.6 ± 13.4	59.5 ± 13.3		63.7 ± 13.1	< 0.001
**Race**, n (%)					< 0.001
Non-Hispanic white	2756 (53.2)	1967 (52.4)		789 (55.5)	
Non-Hispanic black	940 (18.2)	596 (15.9)		344 (24.2)	
Mexican American	1100 (21.3)	900 (24)		200 (14.1)	
Others	380 (7.3)	291 (7.8)		89 (6.3)	
**Education level**(years), n (%)					< 0.001
< 9	1006 (19.4)	764 (20.4)		242 (17)	
9 ∼ 12	2036 (39.3)	1359 (36.2)		677 (47.6)	
> 12	2134 (41.2)	1631 (43.4)		503 (35.4)	
**Marital status**, n (%)					< 0.001
Married or living with a partner	3361 (64.9)	2538 (67.6)		823 (57.9)	
Living alone	1815 (35.1)	1216 (32.4)		599 (42.1)	
**Family income**, n (%)					< 0.001
Low	1393 (26.9)	936 (24.9)		457 (32.1)	
Medium	1946 (37.6)	1355 (36.1)		591 (41.6)	
High	1837 (35.5)	1463 (39)		374 (26.3)	
**Calorie intake** (Kcal/d), Median (IQR)	1794.7 (1312.4,2393.2)	1807.0 (1314.2,2415.3)		1764.0 (1304.5,2327.5)	0.031
**Cholesterol**(mmol/L), Mean (SD)	5.4 ± 1.1	5.4 ± 1.1		5.4 ± 1.0	0.693
**Hypertension**, n (%)	1801 (34.8)	1179 (31.4)		622 (43.7)	< 0.001
**Diabetes**, n (%)	730 (14.1)	478 (12.7)		252 (17.7)	< 0.001
**Coronary heart disease**, n (%)	330 (6.4)	210 (5.6)		120 (8.4)	< 0.001
**Stroke**, n (%)	242 (4.7)	140 (3.7)		102 (7.2)	< 0.001
**Smoking status**, n (%)					< 0.001
Never	2442 (47.2)	1861 (49.6)		581 (40.9)	
Current	1771 (34.2)	1270 (33.8)		501 (35.2)	
Former	963 (18.6)	623 (16.6)		340 (23.9)	
**Alcohol drinking**, n (%)					0.908
Yes	1759 (34.0)	1274 (33.9)		485 (34.1)	
No	3417 (66.0)	2480 (66.1)		937 (65.9)	
**Physical activity**, n (%)					< 0.001
Light	2616 (50.5)	1746 (46.5)		870 (61.2)	
Moderate	1389 (26.8)	1042 (27.8)		347 (24.4)	
Vigorous	1171 (22.6)	966 (25.7)		205 (14.4)	
**Balance disorders**, n (%)	1331 (25.7)	844 (22.5)		487 (34.2)	< 0.001

The univariate logistic analysis demonstrated that sex, age, education level, marital status, family income, calorie intake, hypertension, diabetes, coronary heart disease, stroke, current smoking, alcohol drinking and physical activity were associated with balanced disorders (Supplementary table).

**Fig. 2:**
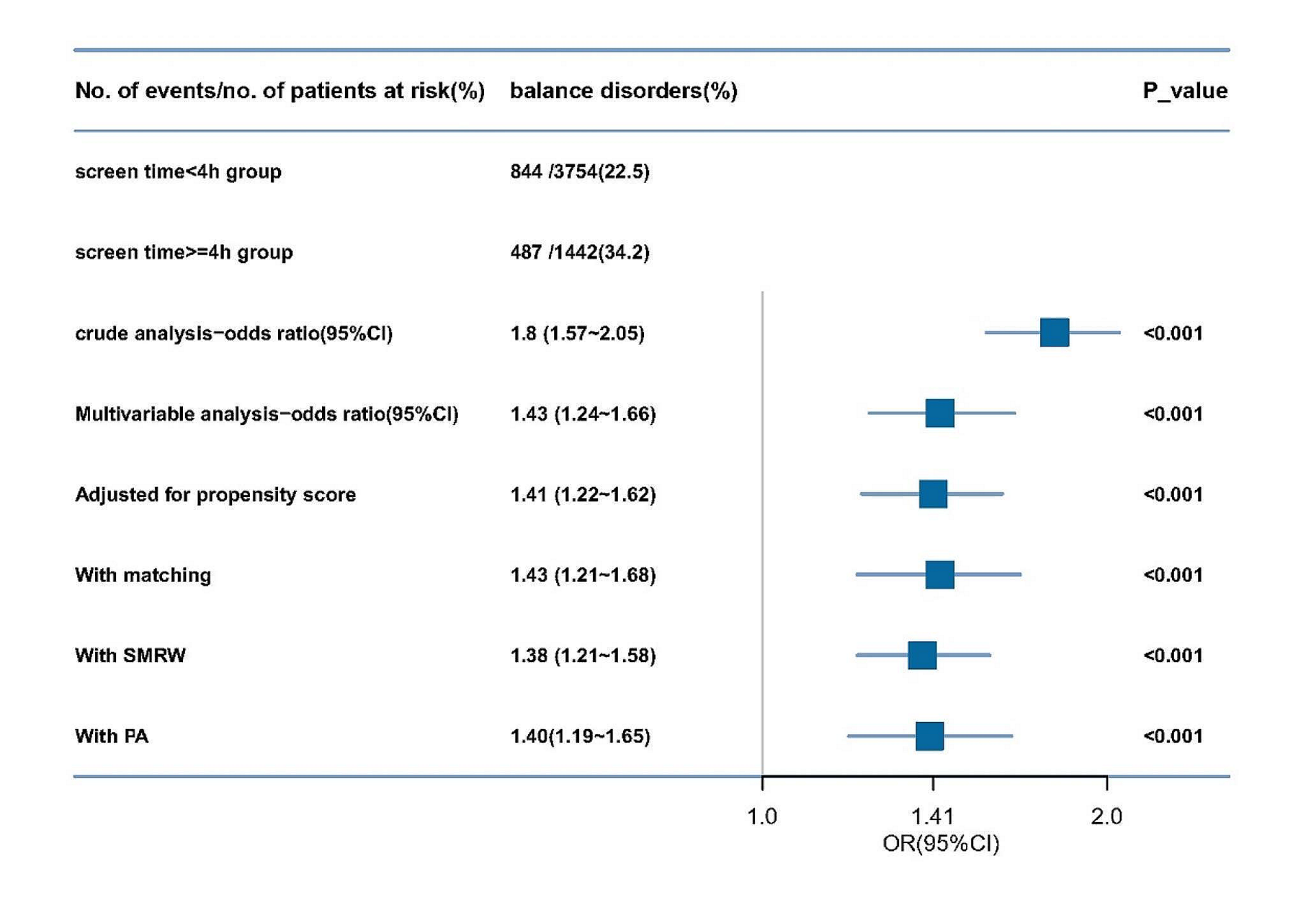
>Forest plot shows ORs of balance disorders risk comparing screen time( ≥ 4 h/d) with this(< 4 h/d) using a variety of models

Multivariate logistic analysis of the unmatched cohort showed significant associated between screen time( ≥ 4 h/d) and balanced disorders OR 1.8 (95%CI 1.57 ∼ 2.05). The results remained stable after adjusting for confounding factors, OR1.43 (95%CI 1.24 ∼ 1.66) (Table 2). Furthermore, the association still remained stable in multivariate analysis using PSM adjusted for propensity score, SMRW and PA. The ORs were 1.38–1.43, all *p* < 0.001 (Fig. 2).

**Fig. 3:**
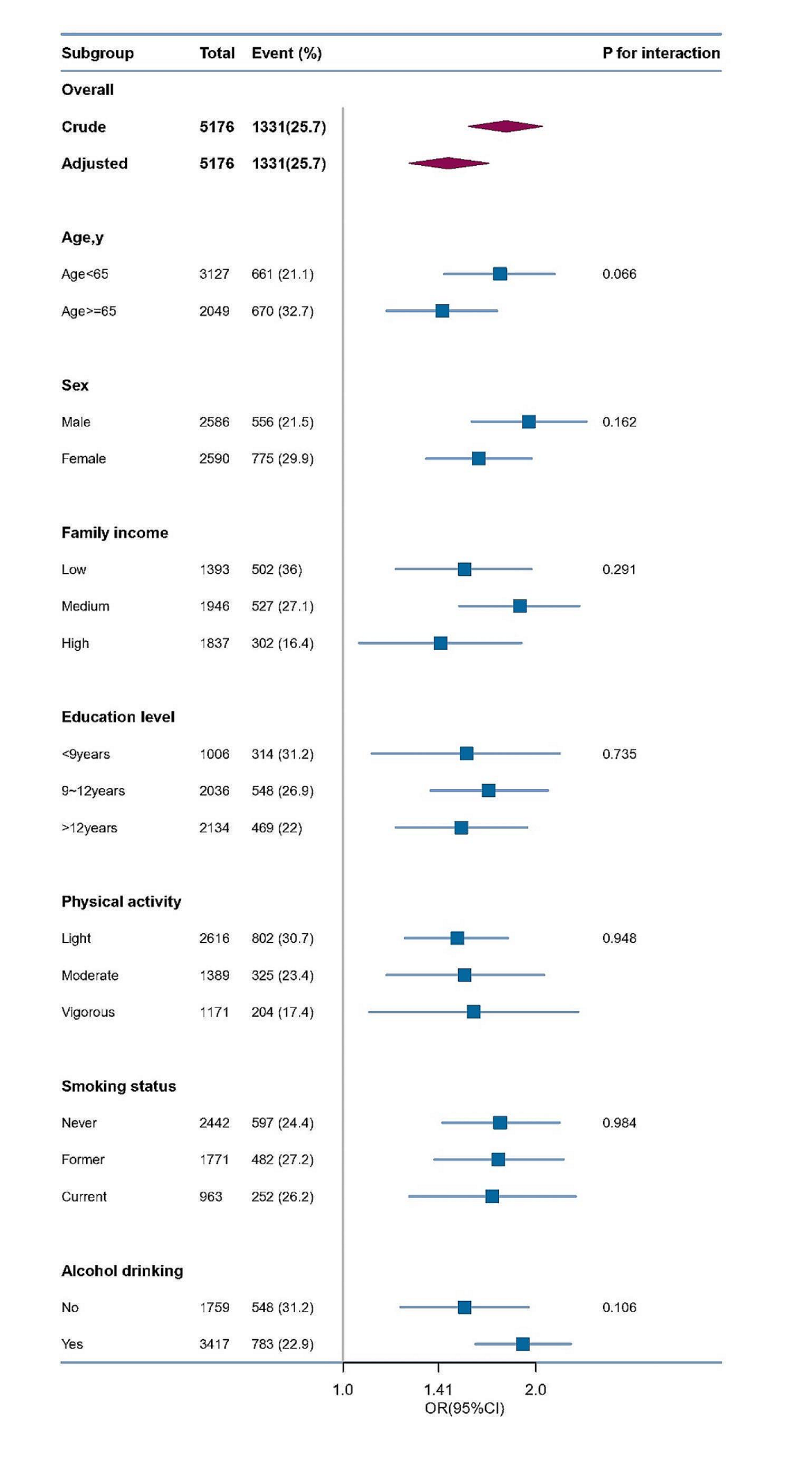
>Forest plot shows ORs of balance disorders risk comparing screen time(  ≥4 h/d) with this(< 4 h/d) using a variety of models in subgroup analysis

Subgroup analysis indicated that the relationship between screen time and balanced disorders was in accordance with other results. No significant interactions were found in any subgroups (Fig. 3).

**Table 2:** >Multivariate logistic analysis of balance disorders risk comparing screen time( ≥ 4 h/d) with this(< 4 h/d)

	screen time (< 4 h/d)	screen time ( ≥4 h/d)	
Model		OR(95%CI)	P value
Unadjusted	1(Ref)	1.8 (1.57 ∼ 2.05)	< 0.001
Model1	1(Ref)	1.57 (1.37 ∼ 1.81)	< 0.001
Model2	1(Ref)	1.49 (1.29 ∼ 1.72)	< 0.001
Model3	1(Ref)	1.46 (1.27 ∼ 1.69)	< 0.001
Model4	1(Ref)	1.43 (1.24 ∼ 1.66)	< 0.001

Model 1 was adjusted for sociodemographic characteristics, including age, sex, race, marital status, education level, and family income

Model 2 was adjusted for sociodemographic characteristics and previous disease (hypertension, diabetes, stroke, and coronary heart disease)

Model 3 was adjusted for sociodemographic characteristics, previous disease, smoking statue and alcohol drinking

Model 4 was adjusted for model3 and calorie intake, total cholesterol, physical activity

## Discussion

Our study found a significant correlation between balance disorders and screen time when the duration of non-work-related screen usage exceeded 4 hours per day, compared to those who used screens for less than 4 hours.

Screen time has emerged as a crucial health indicator in the past decade. Due to changes in work patterns and lifestyles, there has been a notable surge in screen usage [17], thereby exerting a significant impact on both physical and mental well-being, potentially even influencing human cognitive abilities [18]. These effects may be particularly pronounced among middle-aged and elderly individuals due to their declining physical fitness. A cross-sectional study conducted on older Brazilians revealed a positive association between extended screen times and heightened risk of obesity [19]. Ren et al’s investigation demonstrated that individuals aged 40 or above who exceeded two hours of daily screen time experienced a 30% increase in susceptibility to stroke [20]. Furthermore, research involving participants aged 60–88 years discovered that prolonged exposure to screens before bedtime not only correlated with poor sleep quality but also lower family income levels [21]. Evidently, screen time exerts significant influence on the physical and mental well-being of middle-aged and elderly populations. This question is important as excessive screen time has become commonplace and is even regarded as a form of relaxation by many clinicians.

Balance disorders are a prevalent concern among middle-aged and elderly individuals. In our study population, the incidence of balance disorder was found to be approximately 25.7%, aligning closely with previous research findings [1, 22]. Despite advancements in diagnostic techniques, many cases of balance disorders do not respond to singular pharmacological or physical interventions [23]. This is particularly evident among elderly patients due to the multifactorial nature of their condition and the absence of clear etiological specific pathology, thereby significantly escalating healthcare costs and burden [24, 25]. Currently, only a limited number of pilot multidisciplinary interventions for balance problems have been implemented, none of which specifically target the underlying causes [26]. Consequently, it is imperative to investigate its potential etiology.

There are several potential mechanisms that could elucidate the correlation between screen time and dizziness and balance disorders. Firstly, prolonged utilization of screens can induce ocular strain and visual stress, which may impact balance and spatial orientation. Secondly, improper body posture during screen usage can have detrimental effects on bodily equilibrium over extended periods. Additionally, excessive screen exposure may contribute to reduced physical activity levels, which is detrimental to the maintenance of neuromuscular function. Moderately increasing physical activity levels and balance training can not only improve proprioception and neuromuscular function, but also reduce inflammatory factors and prevent bone density loss, helping to improve balance problems [27]. Moreover, the mental manifestations associated with excessive screen time, such as anxiety, have been extensively documented. It is worth noting that anxiety can also contribute to a decrease in levels of physical activity [28]. Furthermore, it has been observed that anxiety can impact eye movement reflexes and gaze stability [29], potentially leading to balance issues. Conversely, individuals suffering from balance disorders often limit their activities to prevent exacerbation of symptoms or further discomfort, inadvertently increasing their screen time. In fact, the interplay between anxiety, dizziness and reduced physical activity is widely acknowledged.

## Limitation

There are certain limitations in this study. Firstly, despite adjusting for confounding factors through various methods, there may still exist potential residual confounders that cannot be entirely eliminated. Secondly, the cross-sectional design employed in this study does not facilitate causal inferences. Additionally, our reliance on self-reported screen time and balance disorders introduces the possibility of recall bias. To address these constraints inherent to observational studies, we anticipate more cohort studies based on device measurements.

## Conclusion

After controlling for all potential confounding variables, the results indicate a substantial link between screen time (which encompasses television viewing, video game playing, and non-work-related computer usage) and impaired balance function among middle-aged and elderly individuals. Nevertheless, it is imperative to corroborate these findings with additional meticulously conducted studies that adhere to rigorous methodologies.

### Electronic supplementary material

Below is the link to the electronic supplementary material.

Supplementary Material 1

## Data Availability

No datasets were generated or analysed during the current study.
